# Clinical comparative analysis of 3D printing-assisted extracorporeal pre-fenestration and Castor integrated branch stent techniques in treating type B aortic dissections with inadequate proximal landing zones

**DOI:** 10.1186/s12872-024-03799-x

**Published:** 2024-02-26

**Authors:** Rongyi Zheng, Huayuan Xi, Fangtao Zhu, Cunwei Cheng, Weihua Huang, Haojie Zhang, Xin He, KaiLin Shen, Ying Liu, QianQian Lu, Haibin Yu

**Affiliations:** The Second Affiliated Hospital of Zhengzhou University, Zhengzhou, China

**Keywords:** Stanford type B aortic dissection, 3D printing, Extracorporeal fenestration TEVAR, Inadequate proximal landing zones, Castor-integrated branch stent

## Abstract

**Background:**

This study aims to compare the clinical effects of two distinct surgical approaches, namely 3D printing-assisted extracorporeal pre-fenestration and Castor integrated branch stent techniques, in treating patients with Stanford type B aortic dissections (TBAD) characterized by inadequate proximal landing zones.

**Methods:**

A retrospective analysis was conducted on 84 patients with type B aortic dissection (TBAD) who underwent thoracic endovascular aortic repair (TEVAR) with left subclavian artery (LSA) reconstruction at our center from January 2022 to July 2023. Based on the different surgical approaches, the patients were divided into two groups: the group assisted by 3D printing for extracorporeal pre-fenestration (*n* = 44) and the group using the castor integrated branch stent (*n* = 40). Clinical indicators: including general patient information, operative time, surgical success rate, intraoperative and postoperative complication rates, re-intervention rate, and mortality, as well as postoperative aortic remodeling, were compared between the two groups. The endpoint of this study is the post-TEVAR mortality rate in patients.

**Results:**

The surgical success rate and device deployment success rate were 100% in both groups, with no statistically significant difference (*P* > 0.05). However, the group assisted by 3D printing for extracorporeal pre-fenestration had a significantly longer operative time (184.20 ± 54.857 min) compared to the group using the castor integrated branch stent (152.75 ± 33.068 min), with a statistically significant difference (t = 3.215, *p* = 0.002, *P* < 0.05). Moreover, the incidence of postoperative cerebral infarction and beak sign was significantly lower in the group assisted by 3D printing for extracorporeal pre-fenestration compared to the castor-integrated branch stent group, demonstrating statistical significance. There were no significant differences between the two groups in terms of other postoperative complication rates and aortic remodeling (*P* > 0.05). Notably, computed tomography angiography images revealed the expansion of the vascular true lumen and the reduction of the false lumen at three specified levels of the thoracic aorta. The follow-up duration did not show any statistically significant difference between the two groups (10.59 ± 4.52 vs. 9.08 ± 4.35 months, t = 1.561, *p* = 0.122 > 0.05). Throughout the follow-up period, neither group experienced new endoleaks, spinal cord injuries, nor limb ischemia. In the castor-integrated branch stent group, one patient developed a new distal dissection, prompting further follow-up. Additionally, there was one case of mortality due to COVID-19 in each group. There were no statistically significant differences between the two groups in terms of re-intervention rate and survival rate (*P* > 0.05).

**Conclusion:**

Both 3D printing-assisted extracorporeal pre-fenestration TEVAR and castor-integrated branch stent techniques demonstrate good safety and efficacy in treating Stanford type B aortic dissection with inadequate proximal anchoring. The 3D printing-assisted extracorporeal pre-fenestration TEVAR technique has a lower incidence of postoperative cerebral infarction and beak sign, while the castor-integrated branch stent technique has advantages in operative time.

## Introduction

With the continuous advancement and refinement of intravascular techniques and medical devices, Thoracic Endovascular Aortic Repair (TEVAR) has emerged as the preferred modality for treating aortic pathologies [1, 2], traditional open surgical approaches for aortic lesions involving arch vessel branches have gradually been phased out due to their associated high trauma, mortality rates, and complication occurrence [3]. In recent years, there has been an increase in the applications of 3D printing-assisted open aortic fenestration techniques and integrated branch stent-graft TEVAR technology, particularly in cases of aortic dissections with proximal anchoring insufficiency [4]. These techniques are used when the distance between the tear and the left subclavian artery (LSA) is less than 15 mm [5] (5), necessitating coverage of the LSA or left common carotid artery (LCCA) [6] (6). However, due to the relatively short clinical application history and limited related clinical literature, debates persist regarding their safety and efficacy [7–9]. Therefore, this study aims to summarize the experiential applications of these two techniques, assess their short-term clinical outcomes in the treatment of proximal anchoring insufficient aortic dissections, and analyze their pros and cons, providing new choices and insights for clinical treatment.

## Methods

### Study design

This is a retrospective, double-blind, randomized clinical trial that collected clinical data from 84 patients with aortic arch descending lesions involving the left subclavian artery (LSA) who underwent TEVAR treatment at our center from January 2022 to July 2023. We employed a randomization principle to group the patients, with preoperative assessment of the patients’ condition by two or more specialist physicians and comprehensive communication with the patients’ family members to obtain their informed consent before adopting an appropriate surgical plan. Based on the different surgical approaches, the patients were divided into Group A and Group B: Group A underwent 3D printing-assisted extracorporeal pre-fenestration stent placement (*n* = 44), while Group B underwent integrated branch stent placement using Castor technology (*n* = 40). This study was conducted in accordance with the Helsinki Declaration and approved by the Ethics Committee of the Second Affiliated Hospital of Zhengzhou University (approval number: 2023225). Informed consent was obtained from all patients before surgery.

### Inclusion criteria

(1) Patients diagnosed with Stanford Type B aortic dissection based on clinical symptoms and CTA examination; (2) CTA measurement of proximal anchoring zone length ≤ 15 mm; (3) CTA measurement of proximal anchoring zone length ≥ 15 mm, with retrograde dissection or hematoma within the zone; (4) Patients who underwent 3D printing-assisted extracorporeal pre-fenestration or castor-integrated branch stent-graft TEVAR surgery; (5) Patients with complete postoperative follow-up data. Exclusion criteria: (1) Patients with severe hepatic or renal dysfunction unsuitable for surgery; (2) Patients with Type A aortic dissection, retrograde dissection, or hematoma involving the left common carotid artery and its proximal segment; (3) Patients with thoracic aortic intramural hematoma, aneurysm, thoracic aortic ulcer; (4) Preoperative CTA showing dissection involving aortic arch branches other than the LSA; (5) Patients who did not undergo 3D printing-assisted extracorporeal pre-fenestration or castor-integrated branch stent-graft TEVAR surgery; (6) Patients with incomplete postoperative follow-up data; (7) Patients with concurrent Marfan syndrome or other connective tissue disorders. All patients underwent preoperative aortic computed tomography angiography (CTA) to confirm the presence of aortic dissections and classify them according to the reference guidelines [1]. There were 25 cases in hyperacute (< 24 h ) and 59 cases in acute ( 1–14 days ). Indications for performing thoracic endovascular aortic repair (TEVAR) at our institution include anatomical suitability, signs of aortic rupture and/or visceral malperfusion, refractory hypertension, and persistent pain in cases of hyperacute, acute, or subacute complicated TBAD. TEVAR surgery is performed for uncomplicated TBAD within approximately 90 days after initial symptoms. The risk factors of patients were shown in Table 1, which were in line with the indications of TEVAR.

### Preoperative preparation and 3D printing model fabrication

**Fig. 1:**
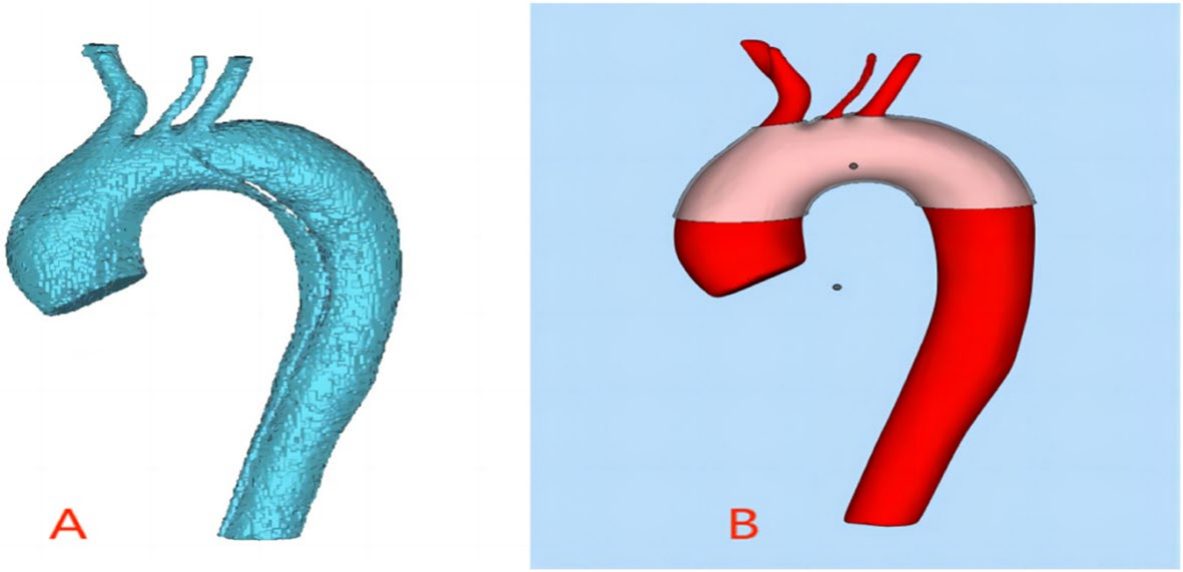
Picture **A**: 3D printing of aortic simulated model Picture **B**: designing fenestrations with 3D printing assistance

Firstly, we imported the original DICOM format CTA data into Endosize software to perform a three-dimensional reconstruction of the aortic arch vessels and measure critical aortic data for surgical planning. Subsequently, we input the CTA data into Mimics 21.0 software to conduct three-dimensional reconstruction of the aortic arch region, including the diseased aortic segment, branching openings, and the normal proximal and distal anchoring zones (see Fig. 1 Picture A). Next, we employed reverse engineering software Geomagic Studio 2014 to model the reconstructed data, yielding CAD mathematical models. Following simulation analysis using Geomagic Design Direct 2014 engineering design software, we designed aortic fenestration templates as per the plan (see Fig. 1 Picture B). Lastly, we employed a 3D printer to manufacture the design results into aortic models, which were sealed and packaged after ethylene oxide sterilization.

#### Stent graft modification

**Fig. 2:**
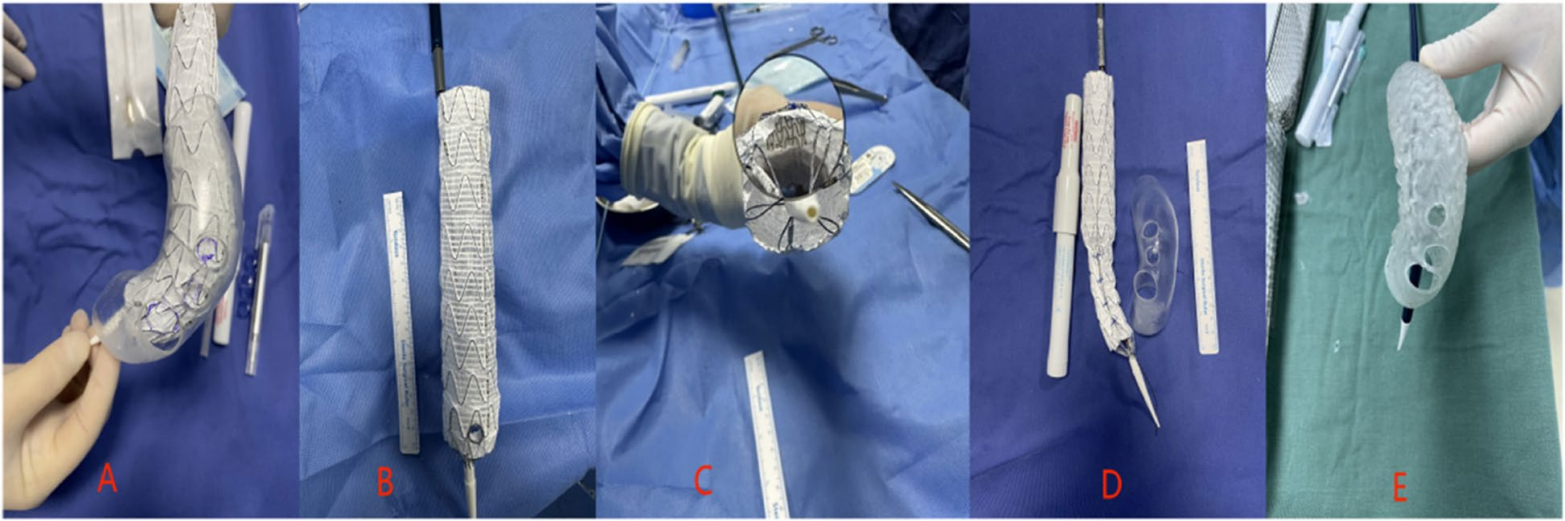
Picture **A**: Within the 3D-printed aortic arch model, the location and dimensions of the primary stent fenestration have been established. Picture **B**: Employing an extracorporeal electrode pen for fenestration. Picture **C**: Suturing the Viabahn stent at the fenestration site Picture **D**: The adjustment of the main stent bundle diameter Picture **E**: Pre-bending of the main stent

According to the preoperative plan, on a sterile operating table, we deployed an appropriately sized aortic-covered stent graft body (Ankura, China) into the sterilized 3D printed model to determine the fenestration position and diameter (See Fig. 2 Picture A). Fenestration was performed using an electrode pen, aiming to create circular fenestration (See Fig. 2 Picture B). We typically used the inlay branch stent graft technique, trimming the Viabahn stent graft (GORE VIABAHN, Gore, USA) to a length of 3–5 mm (diameter less than that of the expected implanted branch stent graft by 1–2 mm). We secured the fenestration area with continuous suturing using 5 − 0 non-absorbable sutures (Prolene, Johnson & Johnson, USA), placing the inner branch inside the large stent graft to prevent internal leakage at the junction (See Fig. 2 Picture C). The LSA bridging stent employed in our procedures predominantly consists of the SilverFlow (Ankura China) system, with a minority of cases utilizing the Viabahn (GORE VIABAHN, Gore, USA) branch stent. Subsequently, we passed a V18 guidewire through the stent graft body at the 6 o’clock position (with the arch apex designated as 12 o’clock) and punctured the delivery sheath to extract one end of the guidewire. We then shrunk the stent graft body (shrinkage proportion at least 30-45%) and secured it onto the guidewire using 5 − 0 Prolene sutures [10], reinserting it into the delivery system after bundling diameter (See Fig. 2 Picture D). Finally, following sterilization of the stent delivery system [11], it was placed inside the stent modified from the 3D printed model, pre-bent to better conform to the curvature of the aortic arch for smooth deployment (See Fig. 2 Picture E). Due to the integrated nature of the Castor integrated branch stent graft, stent-graft modification work was unnecessary.

### Surgical methods

#### 3D- printing group

Following successful patient anesthesia, they were placed in a supine position. A vertical incision was made in the sterilized right groin area, and a segment of the femoral artery was reserved. Both the right femoral artery and left brachial artery were punctured, and 6F femoral sheaths were introduced, followed by the administration of 5000 units of heparin for anticoagulation. A pigtail catheter was introduced to expand the aortic arch, maximizing the exposure of the lesion site. Subsequently, the main stent delivery system was introduced through the femoral artery route. During the advancement of the delivery system, the front handle was secured to prevent rotation [9]. Upon reaching the aortic arch with the main stent, a portion of the stent was partially released to expose the fenestration hole. The diameter of the main support is reduced by 30% ~ 45% by bundling diameter technology, facilitating meticulous fenestration through the LSA while ensuring adequate blood perfusion to the arch branch arteries to minimize the risk of cerebral ischemic events. Once fenestration through the LSA was achieved, long sheaths were individually introduced to deliver the branch-covered stent grafts and were then released. After fully deploying the main stent and removing the tension wire, immediate fluoroscopy was performed to confirm stent coverage of the lesion and ensure unobstructed blood flow in the arch branches. Subsequently, the delivery system was withdrawn, and a balloon was introduced into the branch stent grafts for post-dilation. This was followed by another fluoroscopy to confirm the absence of endoleaks, stent graft narrowing or occlusion, and the patency of branch vessels. Finally, the delivery system was withdrawn, the right femoral artery was closed, and sutures were applied to the various subcutaneous and skin layers.

#### Castor stent group

**Fig. 3:**
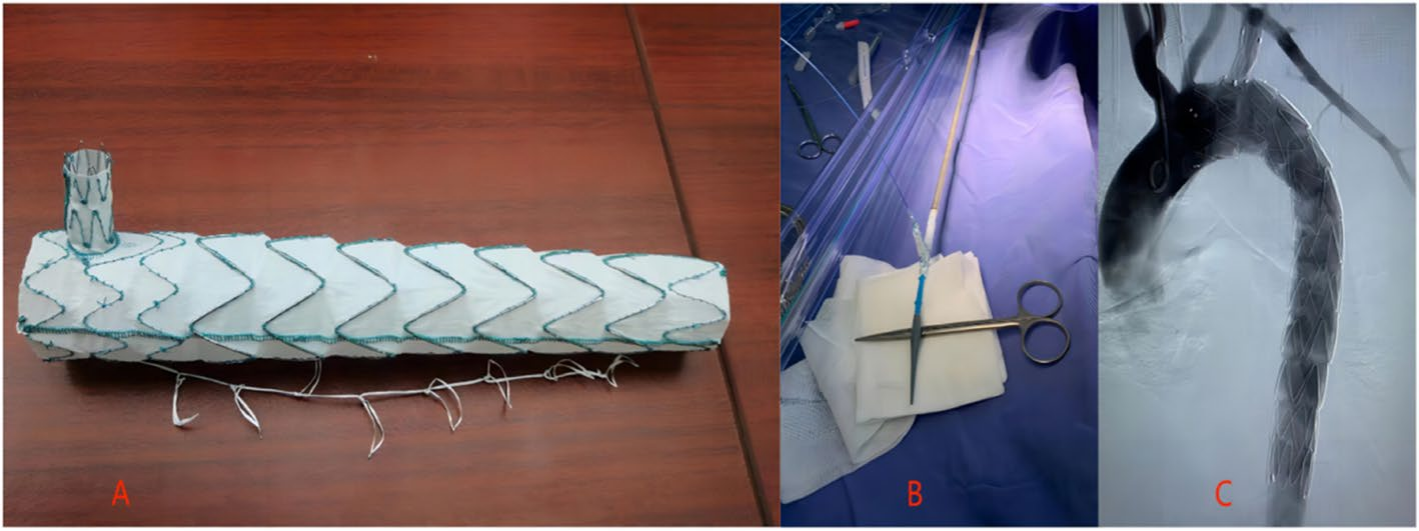
Picture **A**: Castor integrated stent physical image. Picture **B**: Pre-release image of the castor integrated stent. Picture **C**: Post-release radiographic image of the castor integrated stent

Following the successful administration of anesthesia to the patient, they were positioned supine. A 3 cm incision was made in the right groin area, exposing and freeing the right femoral artery. Direct visualization aided the use of an 18G puncture needle to puncture the left brachial artery, leaving a 7F vascular sheath (Cordis) in place. Subsequently, a 5F pigtail catheter (Cordis) was inserted, and angiography was performed to visualize the aortic dissection. Afterward, an 18G arterial puncture needle was used to exchange for a stiff guidewire through the left brachial artery, with the 7F vascular sheath remaining in place. Along the single-curved catheter, an extended guidewire and a 5F single-curved catheter (Cordis, 125 cm) were advanced to the descending aorta and verified to exit from the incision in the right femoral artery. The Castor branched stent graft (See Fig. 3 Picture A) and delivery system (HeartMediTech) were then introduced through the incision in the right femoral artery (See Fig. 3 Picture B), retrieved from the left brachial artery sheath, and guided into the Castor stent along the stiff guidewire with simultaneous traction. This ensured the precise placement of the stent without any entanglement. Following this, controlled blood pressure reduction was initiated, and the stent was released. The Castor branch stent graft constriction membrane was removed through the 7F sheath. To confirm the accurate positioning of the Castor stent, a final angiography was performed (See Fig. 3 Picture C). The main stent was withdrawn, and the branch stent delivery wires were released. Finally, the delivery system was withdrawn, the right femoral artery was closed, and sutures were applied to the subcutaneous and skin layers. Following the surgery, the patient was administered dual antiplatelet therapy for three months. Depending on the patient’s condition, continuation of dual antiplatelet therapy or transition to single antiplatelet therapy may be considered [7].

### Postoperative follow-up and evaluation methods

**Fig. 4:**
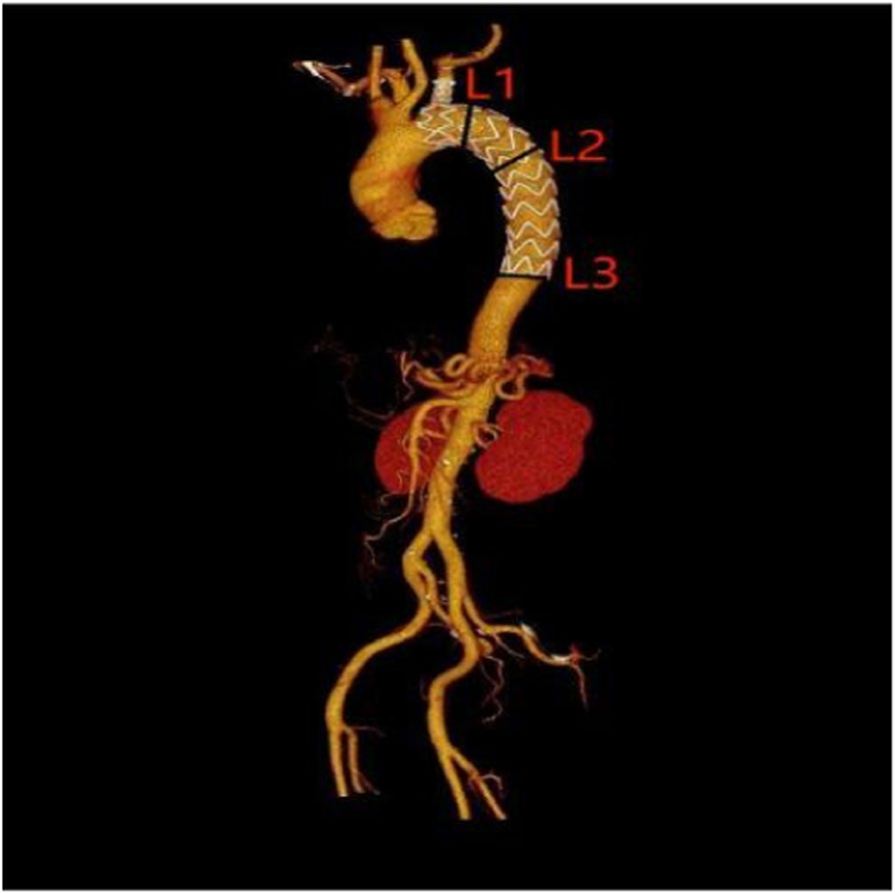
L1: Approximately 1 cm distal to the origin of the LSA at the aortic plane, L2: At the level of the aorta just below the tracheal bifurcation, L3: At the anchoring zone at the distal end of the stent

Postoperative follow-up was conducted through various means, including bedside rounds, telephone inquiries, and outpatient visits, at multiple time points (7–30 days, 3 months, 6 months, and 12 months postoperatively). The observed parameters encompassed surgical success, defined as the precise deployment of the main body stent with adequate sealing of the proximal entry tear during the procedure, as well as intraoperative and postoperative complication rates, rates of secondary surgical interventions, and mortality. Periodic aortic computed tomography angiography (CTA) examinations were performed to assess the patency of the main body stent and branch stents arising from the left subclavian artery (LSA) and to detect the occurrence of endoleak. Measurements were taken at three aortic planes (See Fig. 4), involving the maximal diameter of the vertical intimal flap. Changes in the diameters of the true and false lumens of the aorta, both preoperatively and postoperatively, were compared, along with an assessment of postoperative false lumen thrombosis, thus enabling an evaluation of aortic remodeling.

### Data processing

Statistical analysis of the data was performed using SPSS version 27.0. Normally distributed data were assessed using independent sample t-tests, while non-normally distributed data were analyzed using non-parametric tests (Mann-Whitney U test). Comparative analyses were conducted using chi-square tests or Fisher’s exact test as appropriate. All tests were two-sided, with a significance level set at α = 0.05.

### Patient characteristics

The preoperative age of patients in both groups (x ± s) was( 55.98 ± 11.472 years) in the 3D- printing group and (53.93 ± 9.939 years) in the Castor stent group (t = 0.872, *p* = 0.386). Detailed preoperative patient information and comorbidities are presented in Table 1, showing no statistically significant differences in baseline characteristics between the two groups (*p* > 0.05).

**Table 1:** Preoperative detailed data and complications of patients

Clinical data	3D-Printing group (*n* = 44)	Castor stent group (*n* = 40)	X²	*P*
Male	31	29	0.043	0.839
Complicated hypertension	27	24	0.016	0.898
Persistent pain	19	17	0.004	0.950
Cardiac insufficiency	16	15	0.012	0.914
Coronary heart disease	13	8	1.018	0.313
Diabetes mellitus	9	7	0.119	0.731
Abdominal aortic atherosclerosis	6	8	0.611	0.434
Combined visceral malperfusion	7	7	0.038	0.845
Combined aortic dilatation	3	5	0.264	0.607

### Perioperative complications in both groups (as shown in Table 2)

**Fig. 5:**
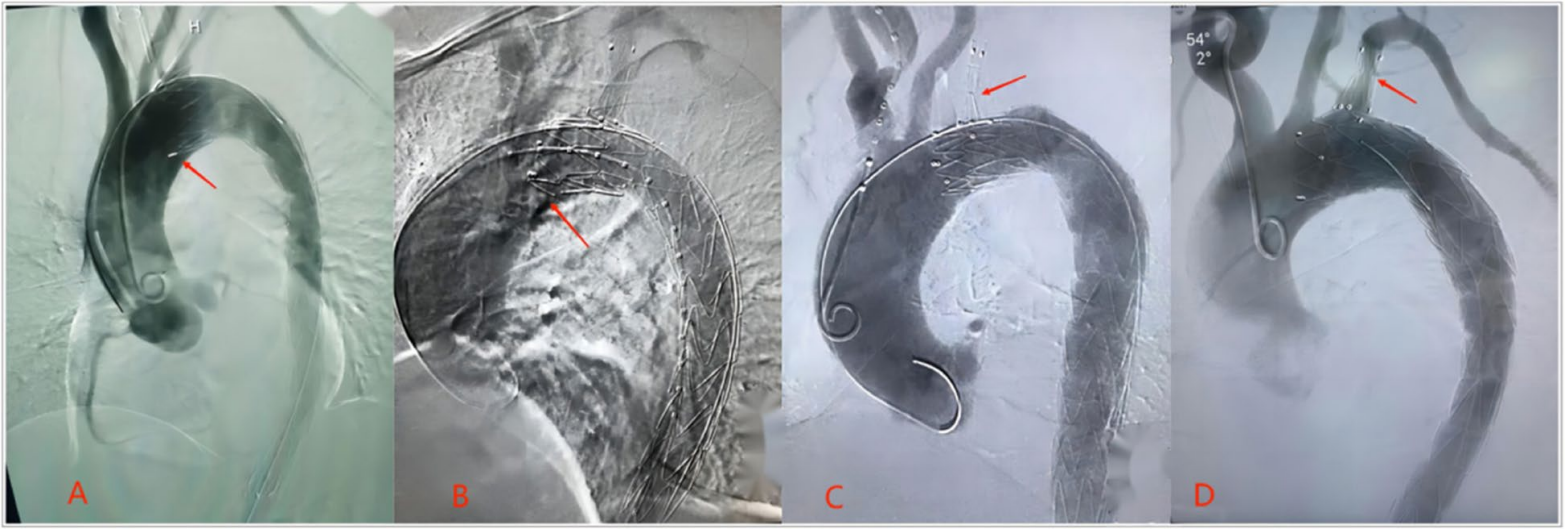
Picture **A**, **B**: Formation of the “Bird-beak sign” at the leading edge of the castor main stent Picture **C**, **D**: Intraluminal narrowing within the Castor branch stents

Both groups achieved a 100% surgical success rate. The 3D-printing group had a significantly longer operative time (184.20 ± 54.857 min) compared to the Castor stent group (152.75 ± 33.068 min), with a statistically significant difference (t = 3.215, *p* = 0.002, *p* < 0.05). However, the incidence of postoperative cavity-type cerebral infarctions (It was revealed by MRI scans of the brains of patients with revealed symptoms of neurological dysfunction after surgery.) and “bird-beak” signs (See Fig. 5 Picture A, B) in the 3D- printing group was significantly lower than that in the Castor stent group, with statistically significant differences. There were no statistically significant differences between the two groups in terms of other complication rates, secondary surgical intervention rates, and mortality (*p* > 0.05).

**Table 2:** Results of postoperative complications

Clinical data	3D-Printing group (*n* = 44)	Castor stent group (*n* = 40)	X²	*P*
Postoperative infection	5	3	0.053	0.818
Postoperative lacunar cerebral infarction	1	7	4.009	0.045
Postoperative pain	3	6	0.736	0.391
Postoperative limb weakness	1	1		1.00
Postoperative endoleak	2	1		1.00
Bird-beak sign	0	6	5.026	0.025

### Follow-up results

There was no statistically significant difference in the follow-up duration between the two groups (10.59 ± 4.52 vs. 9.08 ± 4.35 months, t = 1.561, *p* = 0.122). During the follow-up period, neither group experienced new endoleaks, spinal cord injuries, or limb ischemia. In the Castor stent group, one patient developed a new dissection in the distal part of the stent and continued to be observed during follow-up. Additionally, one patient from each group died from COVID-19. There were no statistically significant differences in secondary surgical intervention rates and survival rates between the two groups (*p* > 0.05).

### Comparison of aortic remodeling after dissection in both groups (as shown in Tables 3 and 4)

We collected preoperative and postoperative (within one month) thoracoabdominal aortic CTA data for both groups and conducted three-dimensional reconstruction and measurements using Endosize. The results are presented in Table 4: (1) In both groups, there was a significant increase in true lumen expansion and a significant decrease in false lumen expansion postoperatively (at the L1, L2, and L3 levels), with statistically significant differences (*p* < 0.05). (2) In the stented segments (at the L1, L2, and L3 levels), there were no significant differences between the 3D-printing group and the castor stent group in terms of true lumen diameter expansion rate and the degree of thrombosis in both stented and non-stented segments (*p* > 0.05). However, there was a statistically significant difference in the degree of thrombosis in the intrinsic false lumen, with the stented segment being significantly better than the non-stented segment (X²=5.390, 4.878, *p* = 0.02, 0.027, *p* < 0.05).

**Table 3:** Two groups of different aortic plane true and false lumen diameter change rate

Plane	Change rate	3D-Printing group (*n* = 44)	Castor stent group (*n* = 40)	*P*
L1	R(DTL)	55.90 ± 26.77	66.79 ± 45.71	0.191
	R(DFL)	-71.53 ± 25.59	-69.20 ± 22.92	0.5
L2	R(DTL)	72.64 ± 35.49	76.97 ± 69.04	0.936
	R(DFL)	-78.69 ± 22.17	-70.34 ± 24.30	0.125
L3	R(DTL)	69.82 ± 34.32	68.36 ± 46.76	0.531
	R(DFL)	-71.40 ± 31.34	-71.99 ± 23.47	0.49

R(DTL) is the rate of true lumen diameter expansion, R(DFL) is the rate of false lumen diameter expansion, rate of true and false lumen diameter expansion = (postoperative true and false lumen diameter – preoperative true and false lumen diameter) / preoperative true and false lumen diameter × 100%

**Table 4:** The degree of thrombosis in the false lumen of thrombus in the two groups

site	Degree of false lumen thrombosis	3D-Printing group (*n* = 44)	Castor stent group (*n* = 40)	X²	*P*
Aortic stent segment	Complete thrombosis or disappearance of false lumen	32	29	0.024	0.988
	Partial thrombosis	8	7		
	No thrombosis	4	4		
Aortic non-stent segment	Complete thrombosis or disappearance of false lumen	10	7	0.546	0.761
	Partial thrombosis	22	23		
	No thrombosis	12	10		

## Discussion

For patients with aortic dissections who are not suitable candidates for open surgery, TEVAR serves as a safer and more reliable alternative. However, a healthy proximal anchoring zone of at least 15 millimeters is typically required for the safety and stability of the implanted aortic stent graft in TEVAR treatment [5]. Unfortunately, over 40% of aortic dissection patients have inadequate proximal anchoring due to tears less than 15 millimeters from the left subclavian artery (LSA) [12]. Traditional cardiovascular surgical interventions necessitate the administration of general anesthesia followed by a midline thoracic incision. Cardiopulmonary bypass is then initiated to facilitate meticulous examination of the aortic arch and its branches [13], aiming to identify the precise location of the intimal tear and ascertain the extent of the lesion. Subsequently, an optimal surgical approach is chosen based on these findings. Nevertheless, owing to the substantial trauma and increased incidence of postoperative complications associated with traditional surgical techniques [14], Tevar has emerged as a viable alternative in recent years. The debranching hybrid surgery is commonly employed to extend the proximal anchoring zone [15]. Common procedures include carotid-subclavian artery bypass, subclavian-carotid transposition (end-to-side anastomosis), and carotid-axillary artery bypass combined with TEVAR. However, it still requires high-risk, highly invasive open chest surgery. Chimney stent techniques and in-situ fenestration techniques are also frequently used for LSA reconstruction; however, both methods require temporary coverage of the arch branches, which increases the risk of stroke [16]. Furthermore, chimney stent techniques have a higher incidence of late endoleaks due to the formation of grooves between parallel stents and the main stent [17]. Extracorporeal fenestration combined with TEVAR technology has been widely adopted for LSA reconstruction due to its acceptable technical success rate and complication rate. Nevertheless, it is important to note that precise stent fenestration positioning, fenestration diameter, and accurate alignment with the LSA opening are critical factors in extracorporeal fenestration technology, and the anatomical complexity of the aortic arch further complicates this technique [18].

With the rapid advancement of 3D printing technology, its application in the medical field has become increasingly extensive. Studies have demonstrated the accuracy and reliability of 3D-printed aortic models, which can effectively guide extracorporeal fenestration surgery for aortic stents [19, 20]. In comparison to traditional auxiliary examination methods like CT scanning and CT three-dimensional reconstruction, 3D printed models provide more intuitive and precise data, thereby playing a crucial role in surgical planning and stent selection. 3D-printed aortic arch models offer two distinct advantages in aortic stent reconstruction [8, 21]. Firstly, the hollow and transparent nature of these models allows for precise fenestration positioning within the model after stent graft deployment, reducing potential errors caused by manual measurements. Secondly, the accurate replication of the aortic arch location enables the aortic stent graft to more accurately simulate the post-implantation position and the position of branch vessel openings compared to measurements solely obtained from CTA. This facilitates a more precise simulation of the spatial relationship between the aortic stent graft and the arch branches. In cases of aortic anatomical variations, such as bovine arch, vagal subclavian artery, left vertebral artery originating from the aortic arch, or a highly twisted or curved aortic arch, these anatomical conditions can increase the difficulty of extracorporeal fenestration surgery [22]. Therefore, utilizing 3D printing technology to create anatomically complex aortic arch models not only allows for direct visualization of the lesion’s anatomy but also enables the simulation of the twisting of the aortic stent graft. This, in turn, facilitates more accurate fenestration design, reduces surgical difficulty, and ensures safety. Moreover, apart from guiding physicians in aortic stent fenestration surgery, 3D-printed models can visually display aortic morphology and the anatomical relationships of various branch arteries. This aids in enhancing understanding of the disease and surgical plans for both physicians and patients, promoting doctor-patient communication, and improving young physicians’ knowledge of aortic diseases [23]. Furthermore, in precise fenestration procedures guided by 3D-printed models, our center combines the use of the “bundle diameter” technique. This technique involves reducing the diameter of the main stent by at least 30–45% before reinserting it into the delivery system, allowing for a more extensive in-vessel adjustment range. Compared to traditional extracorporeal fenestration techniques, this approach ensures sufficient blood flow to the branch arteries, reducing the likelihood of cerebral ischemic events. Additionally, it facilitates easier super-selection of the branch artery guidewire into the fenestration, thereby reducing the alignment time between the stent fenestration location and branch arteries, as well as the stent deployment time. Consequently, this shortens the surgical duration and lowers the incidence of postoperative complications [24, 25]. Additionally, the intrabranch technique involves suturing an appropriately sized intrabranch stent at the site of the large stent fenestration. This transforms the original “wire-to-surface” contact into “surface-to-surface” contact, effectively preventing the occurrence of type I endoleaks associated with traditional extracorporeal fenestration techniques [26]. Furthermore, the integrated design of the Castor stent eliminates the need for assembly and mitigates the risk of stent dislocation due to assembly-related leaks. The connection between the branch segment and the graft main body is composed of flexible polyester fabric, enabling the branch segment to be easily drawn into the intended branch artery. The branch-to-main stent junction offers a 150-degree rotational range, accommodating the typical angles between branch arteries and aortic arch stents [27]. Currently, at our center, both 3D-printing-assisted extracorporeal pre-fenestration techniques and Castor integrated branch techniques are widely employed in treating TBAD with inadequate proximal anchoring zones.

Our study compared the short and mid-term clinical outcomes of patients with TBAD and inadequate proximal anchoring zones treated with these two surgical approaches. The baseline data, surgical success rate, secondary intervention rate, and mortality showed no statistically significant differences between the two groups (*p* > 0.05). These findings indicate that both approaches are safe and effective, consistent with previous domestic and international studies [28, 29]. However, we observed significantly lower postoperative rates of cavity-type cerebral infarctions and “bird-beak” signs in the 3D-printing-assisted extracorporeal fenestration group compared to the castor stent group (*p* < 0.05). Several factors may contribute to this disparity. Firstly, the branch segments of the Castor integrated branch stent are relatively flexible, making them prone to distortion in cases where the angle between the left subclavian artery (LSA) and the aortic arch is small. This distortion can lead to the narrowing of the branch stent [30] (See Fig. 5 Picture C, D), slowed blood flow, and the formation of small thrombi, which increase the risk of lacunar cerebral infarction. In contrast, the 3D-printing-assisted extracorporeal fenestration approach utilizes a separate metal-covered branch stent inserted through the brachial artery into the main stent fenestration below the LSA. This technique better adapts to vascular anatomical structures, resulting in a significantly lower probability of stent distortion and branch stent narrowing. Secondly, the Castor integrated branch stent lacks a stent-covered area at its main front end and has only a short stent-covered area. In cases of aortic arches with significant curvature or complex morphologies, “bird-beak” signs are more likely to occur, impeding stent anchoring and increasing the risk of stent migration. Additionally, the castor stent group had a significantly shorter operation time compared to the 3D-printing-assisted extracorporeal fenestration group (*p* < 0.05). This is mainly because the Castor integrated branch stent does not require extracorporeal stent modification, resulting in a lower rate of endoleakage [31]. However, in our study, there was no significant difference in the postoperative endoleak rate between the two groups. Further studies with larger sample sizes are needed to confirm these findings. Lastly, the expansion rate of the true and false lumens and the degree of thrombosis after TEVAR are reliable indicators for evaluating the long-term prognosis of TBAD patients [32, 33]. In our study, we found no significant differences between the two groups in terms of the expansion rate of the true and false lumens and the degree of thrombosis between stented and non-stented segments. However, within each group, the postoperative expansion rate of the true lumen and the degree of false lumen thrombosis in the stented segment were significantly better than those in the non-stented segment (*p* < 0.05). This suggests that stented segments have a better effect on aortic remodeling [34, 35]. 

### Limitations

(1) Currently, the materials used in 3D-printed aortic models are relatively rigid and cannot fully simulate the changes in the aorta under force [36, 37]. With advancements in 3D printing technology, we anticipate obtaining more flexible and elastic materials that can accurately simulate the physiological conditions of the aortic arch. Additionally, we are exploring ways to shorten the model preparation time to benefit critically ill patients requiring urgent surgery. (2) The Castor integrated branch stent has some major limitations: ① The current stent models may not meet the needs of all cases due to individual anatomical variations, particularly in reconstructing double-branch or triple-branch scenarios. ② The release steps are relatively complex and require sufficient release space to expand the branch stent, which may carry a higher risk of complications such as arterial wall injury, plaque, or thrombus detachment leading to lacunar cerebral infarction. However, we acknowledge that our study has limitations, including a small sample size, a short follow-up period, and the need for more data to assess medium to long-term treatment outcomes [38]. Therefore, larger-scale, longer-term, and more rigorous randomized controlled trials are urgently needed to validate the efficacy and feasibility of 3D-printing-assisted extracorporeal pre-fenestration techniques in treating TBAD patients with inadequate proximal anchoring zones.

## Conclusion

In summary, for treating standard type B aortic dissections with inadequate proximal anchoring zones, both of these techniques demonstrate good safety and effectiveness. For TBAD patients with complex aortic arch anatomy (Type II and Type III), the 3D-printing-assisted extracorporeal pre-fenestration TEVAR technique exhibits a lower postoperative incidence of cavity-type cerebral infarctions and “bird-beak” signs, while the Castor integrated branch stent technique offers an advantage in terms of shorter operative times, particularly suited for cases with relatively simple aortic arch anatomy, critically ill patients requiring urgent surgery.

## Data Availability

Datasets used and/or analyzed for this study are available from the corresponding author upon appropriate request.

## Abbreviations

CTA: Computed tomography angiography
LSA: Left subclavian artery
TEVAR: Thoracic endovascular aortic repair
TBAD: Type B aortic dissection
LCCA: Left common carotid artery
3D-printing group: 3D printing-assisted extracorporeal pre-fenestration group
Castor stent group: Castor- Integrated branch stent group
